# Selinexor‐based regimens in patients with multiple myeloma after prior anti‐B‐cell maturation antigen treatment

**DOI:** 10.1002/jha2.572

**Published:** 2022-09-30

**Authors:** Muhamed Baljevic, Cristina Gasparetto, Gary J. Schiller, Sascha A. Tuchman, Natalie S. Callander, Suzanne Lentzsch, Jorge Monge, Rami Kotb, Nizar J. Bahlis, Darrell White, Christine I. Chen, Heather J. Sutherland, Sumit Madan, Richard LeBlanc, Michael Sebag, Christopher P. Venner, William I. Bensinger, Noa Biran, Andrew DeCastro, Dane R. Van Domelen, Chris Zhang, Jatin J. Shah, Sharon Shacham, Michael G. Kauffman, Ohad S. Bentur, Brea Lipe

**Affiliations:** ^1^ Vanderbilt‐Ingram Cancer Center Vanderbilt University Medical Center Nashville Tennessee USA; ^2^ Duke Cancer Institute Duke University Durham North Carolina USA; ^3^ Hematological Malignancy/Stem Cell Transplant Program University of California ‐ Los Angeles David Geffen School of Medicine Los Angeles California USA; ^4^ Department of Medicine Division of Hematology The University of North Carolina at Chapel Hill Chapel Hill North Carolina USA; ^5^ Division of Hematology/Oncology, Department of Medicine University of Wisconsin‐Madison School of Medicine and Public Health Madison Wisconsin USA; ^6^ Columbia University Medical Center New York New York USA; ^7^ Weill Cornell Medicine New York New York USA; ^8^ Medical Oncology and Hematology CancerCare Manitoba Winnipeg Manitoba Canada; ^9^ Arnie Charbonneau Cancer Research Institute University of Calgary Calgary Alberta Canada; ^10^ Department of Medicine/Division of Hematology Dalhousie University Halifax Nova Scotia Canada; ^11^ Princess Margaret Cancer Centre Toronto Ontario Canada; ^12^ Division of Hematology Vancouver General Hospital Vancouver British Columbia Canada; ^13^ Banner MD Anderson Cancer Center Gilbert Arizona USA; ^14^ Maisonneuve‐Rosemont Hospital University of Montreal Montreal Québec Canada; ^15^ Division of Hematology McGill University Health Centre Montreal Québec Canada; ^16^ Cross Cancer Institute University of Alberta Edmonton Alberta Canada; ^17^ Center for Blood Disorders and Stem Cell Transplantation Swedish Cancer Institute Seattle Washington USA; ^18^ John Theurer Cancer Center, Hackensack Meridian Health Hackensack University Medical Center Hackensack New Jersey USA; ^19^ Karyopharm Therapeutics Inc. Newton Massachusetts USA; ^20^ Wilmot Cancer Institute University of Rochester Medical Center Rochester New York USA

**Keywords:** anti‐BCMA, multiple myeloma, selinexor

## Abstract

There is a lack of consensus on therapy sequencing in previously treated multiple myeloma, particularly after anti‐B‐cell maturation antigen (BCMA) therapy. Earlier reports on selinexor (X) regimens demonstrated considerable efficacy in early treatment, and after anti‐BCMA‐targeted chimeric antigen receptor‐T cell therapy. Here, we present data from 11 heavily pretreated patients who predominantly received BCMA‐antibody‐drug conjugate therapy. We observe that X‐containing regimens are potent and achieve durable responses with numerically higher overall response and clinical benefit rates, as well as median progression free survival compared to patients’ prior anti‐BCMA therapies, despite being used later in the treatment course. In an area of evolving unmet need, these data reaffirm the efficacy of X‐based regimens following broader anti‐BCMA therapy.

1

Multiple myeloma (MM) is an incurable hematologic malignancy characterized by remissions and relapses with multiple lines of therapy followed by the development of refractoriness and death. Immunomodulatory agents (IMiDs), proteasome inhibitors (PIs), and anti‐CD38 monoclonal antibodies (mAbs) are typically used in first‐ and second‐line treatment. Upon becoming refractory to those classes of agents (i.e., triple‐refractory MM), newer agents that work through non‐cross‐resistant mechanisms can induce remissions, which are generally short‐lived with relatively poor clinically meaningful outcomes [1, 2, 3, 4]. Selinexor (X), an oral first‐in‐class selective inhibitor of nuclear export (SINE) compound that inhibits exportin‐1(XPO1), forcing nuclear retention and reactivation of tumor suppressor proteins, demonstrates clinical benefit and objective responses in patients with first or later‐line relapses, including those with triple class refractory MM [5, 6]. Separately, cellular chimeric antigen receptor T cell (CAR‐T) therapy (CAR‐T) and antibody‐drug conjugate (ADC)‐based agents targeting B‐cell maturation antigen (BCMA) have shown activity in heavily pretreated MM [7].

Although the list of options is broad, establishing maximally effective sequence of deploying these different regimens remains a challenge, with no clear consensus. There is even less clarity on managing anti‐BCMA previously treated MM, representing an evolving area of unmet need given the increased study of upfront CAR‐T‐based therapies [8, 9].

Selinexor is approved for use with low‐dose dexamethasone in heavily pretreated MM or with bortezomib and dexamethasone (XVd) in patients with ≥1 prior therapy [5, 6]; other combinations with IMiDs, PIs and anti‐CD38 mAbs are also listed in national guidelines [10]. Recent additions approved in MM after ≥4 lines of prior therapies (including an IMiD, a PI and an anti‐CD38 mAb), include CAR‐T agents idecabtagene vicleucel (ide‐cel) and ciltacabtagene autoleucel, and the anti‐BMCA ADC belantamab mafodotin.

Since BCMA‐directed agents are relatively new, outcomes in MM previously treated with anti‐BCMA agents are relatively unknown. Combinations of drugs with selinexor have shown strong clinical benefit in heavily pretreated MM, including MM refractory to anti‐BCMA CAR‐T therapy. In a previous report of CAR‐T pretreated MM, X‐containing regimens led to objective responses in seven patients with one stringent complete response, three very good partial responses (VGPRs), and two partial responses (PRs) (overall response rate [ORR] 85.7%, and clinical benefit rate [CBR] 100% with one additional minor response [MR]) [11]. Importantly, a recent report of non‐X‐containing treatment outcomes in seven patients with MM refractory to ide‐cel showed an ORR to the first subsequent therapy of 28.5% (one VGPR and one PR), a CBR of 57.1% (one additional MR, and one stable disease [SD], and median progression‐free survival [PFS] of 2 months) [12].

Although selinexor is approved for use in patients with ≥1 prior therapy in combination with bortezomib and dexamethasone, to better understand the effect of X‐containing regimens in patients with heavily pretreated MM, particularly in those previously treated with anti‐BCMA agents not limited to CAR‐T, we evaluated the responses to therapy with selinexor post‐anti‐BCMA therapy in the selinexor and backbone treatments of multiple myeloma patients (STOMPs) study. Here, we report treatment outcomes for eleven new patients from STOMP who were previously treated with anti‐BCMA agents, including seven who received an anti‐BCMA ADC.

STOMP is a multicenter, open‐label, phase 1b/2 clinical study designed to assess the efficacy and safety of 10 combination therapies of selinexor with backbone agents in 11 study arms in patients with previously treated or newly diagnosed MM. The study is ongoing in the US and Canada (ClinicalTrials.gov NCT02343042). The CBR was defined as ORR plus minimal response. Minimal response was defined by the 2016 IMWG response criteria as >25% but <49% reduction of serum M‐protein and reduction in 24‐hr urine M‐protein by 50%–89%. In addition to the above criteria, if present at baseline, a ≥50% reduction in the size of soft tissue plasmacytomas was also required [13].

Eleven patients who received prior anti‐BCMA therapy were treated with five different selinexor‐containing regimens, including nine (81.8%) with three triplets and two (18.2%) with two quadruplet regimens (Table 1). Median age was 71 years (range 46–85), seven patients (63.6%) were women, and all were white. Median duration from MM diagnosis to treatment with a STOMP regimen was 6.9 years (range 2.3–12.8) and patients received a median of 6 prior lines of therapy (range 4–10). Eight patients (72.7%) received anti‐BCMA therapy as immediate prior therapy before STOMP. Among 10 patients (90.9%), selinexor was the only new drug in the treatment regimen. Six patients (54.5%) had high‐risk disease, defined via high‐risk cytogenetics or extramedullary disease at screening.

**TABLE 1:** Baseline characteristics and response to treatments

**Feature**	**Patients with ADC‐BCMA pretreatment** **^a^**							**Patients without ADC‐BCMA pretreatment**			
**Patient number**	**1**	**2**	**3**	**4**	**5**	**7**	**10**	**6**	**8**	**9**	**11**
**STOMP treatment**	XVd	XVd	XVd	XPd	XPd	XKd	XPd	XPd	XPVd	XPEd	XKd
**ISS stage at initial diagnosis**	I	Unknown	III	II	I	II	I	Unknown	III	II	Unknown
**Diagnosis to 1st STOMP dose (yr)**	5.2	6.3	12.8	8.4	6.9	5.7	7.8	4.8	2.3	8.5	8.7
**High risk at diagnosis/Screening** **^b^**	N/N	N/N	N/Y	N/Y	N/N	N/Y	Y/Y	N/N	Y/N	N/N	N/Y
**Number of prior lines of therapy**	5	6	8	10	9	6	4	5	5	7	5
**Prior MM therapies (type)**	V,R,P	V,K,R,P	V,R,P,D (Quad)	V,K,R,P,D (Penta)	V,K,R,P,D (Penta)	V,K,R,P,D (Penta)	V,R,D (Triple)	V,K,N,R,P,D (Penta)	V,K,R,P,D (Penta)	V,K,R,P,D (Penta)	V,K,N,T,R,PD (Penta)
**Refractory MM therapies (type)** **^c^**	R	V,K,R,P	V,R,P	V,K,R,P,D (Penta)	K,R,P,D (Quad)	P,D	V,D	K,N,R,P	K,R,P,D (Quad)	K,R,P,D (Quad)	R,P,D
**Prior ASCT**	Y	Y	Y	Y	N	Y	Y	Y	N	Y	Y
**Anti‐BCMA in immediate prior line**	Y	Y	N	N	Y	Y	Y	Y	Y	N	Y
**Most recent anti‐BCMA therapy**	belantamab mafodotin	belantamab mafodotin	belantamab mafodotin	belantamab mafodotin	MEDI2228	belantamab mafadotin + pembrolizumab	belantamab mafodotin	idecabtagene vicleucel ± daratumumab	SEA‐BCMA ± dex	BCMA BiTE	idecabtagene vicleucel
**Duration of most recent prior anti‐BCMA regimen (mo)**	1.5	0.8	1.1	4.3	1.7	1.4	24.9	2.7	1.4	1.7	5.8
**Best response on most recent prior anti‐BCMA**	PR	SD	SD	VGPR	PD	SD	VGPR	PR	SD	Unknown	PR
**Time from end of anti‐BCMA to 1^st^ STOMP dose (mo)**	0.8	1.0	7.4	35.2	0.2	1.1	1.0	1.3	1.0	14.2	2.6
**Best response on STOMP**	PR	SD	MR	SD	PR	PR	PR	VGPR	PR	MR	VGPR
**Duration of STOMP treatment (mo)**	7.9	6.0	8.1	1.4	2.9	15.8^d^	12.2^d^	15.1	12.9	1.4	13.1^d^

Abbreviations: ADC, antibody‐drug conjugates; ASCT, autologous stem cell transplant; BCMA, B‐cell maturation antigen; D, daratumumab; dex, dexamethasone; IMiD, immunomodulatory drug; ISS, International Staging System; K, carfilzomib; MM, multiple myeloma, mAb, monoclonal antibody; mo, months; MR, minimal response; N, ixazomib; P, pomalidomide; PD, progressive disease; PI, proteasome inhibitor; PR, partial response; R, lenalidomide; SD, stable disease; STOMP, Selinexor and backbone treatments of multiple myeloma patients; T, thalidomide;V, bortezomib; VGPR, very good partial response; XKd, selinexor + carfilzomib + dex; XPd, selinexor + pomalidomide + dex; XPVd, selinexor + pomalidomide + bortezomib + dex; XVd, selinexor + bortezomib + dex; XPEd, selinexor + pomalidomide + elotuzumab + dex; yr, years.

^a^ Patients #1‐5, 7, and 10 were treated with ADC anti‐BCMA therapies.

^b^ High‐risk MM includes the presence of del(17p), t(4;14), t(14;16), gain 1q, or extramedullary myeloma at screening.

^c^ Prior therapy/Refractory therapy type: triple, MM treated with or refractory to PI, IMiD, anti‐CD38 mAb; Quad, MM treated with or refractory to 2 PI, 1 IMiD, and 1 anti‐CD38 mAb OR to 1 PI, 2 IMiD, and 1 anti‐CD38 mAb; Penta, MM refractory to ≥2 PI, ≥2 IMiD, ≥1 anti‐CD38 mAb.

^d^ Ongoing therapy at data cutoff.

The ORR and CBR for the prior anti‐BCMA‐containing regimens were both 50.0% (one patient had an unknown response): two patients had VGPR, three PR, four SD, one progressive disease (PD). Median PFS was 2.0 months (95% CI: 1.5‐ not reached [NR]), and 6‐month PFS probability was 12.0% (95% CI: 1.9–74.4).

As of March 1, 2022, the ORR and CBR for X‐based treatments used *after* anti‐BCMA therapy in this cohort were 63.6% and 81.8%, respectively: two VGPR, five PR, two MR, and two SD; there were no cases of PD as a best response. Median duration of response was not reached (95% CI: 10.6‐NR), but the majority of responses were >6 months (5/7; three are still on therapy without progression) and up to 15.6 months. Median PFS was not reached (95% CI: 6.0‐NR) with median follow‐up of 14.3 months; 6‐month PFS probability was 75.0% (95% CI: 50.3–100.0). Median overall survival (OS) was 14.8 months (95% CI: 10.5‐NR) and median time‐to‐discontinuation was 8.4 months (95% CI: 6.1‐NR), and 8.1 months (95% CI: 3.0‐NR) for the seven patients pretreated with ADC BCMA therapies.

Three patients were still receiving an X‐containing regimen at data cutoff, all with high‐risk disease (Figure 1). Best response was PR in two patients and VGPR in one patient, with duration of response 12.2 months. Six additional patients had >50% reduction in MM tumor burden, including one patient with MR (but with unconfirmed PR based on a single instance of reduction in M‐protein). Median time to response for patients with ≥PR was 1.9 months (95% CI 1.8‐NR), and median time to *any* response (i.e., MR or better) was 1.0 months (95% CI: 1.0‐NR), consistent with rapid onset of anti‐MM activity in the X‐based regimens [5, 14, 15].

**FIGURE 1:**
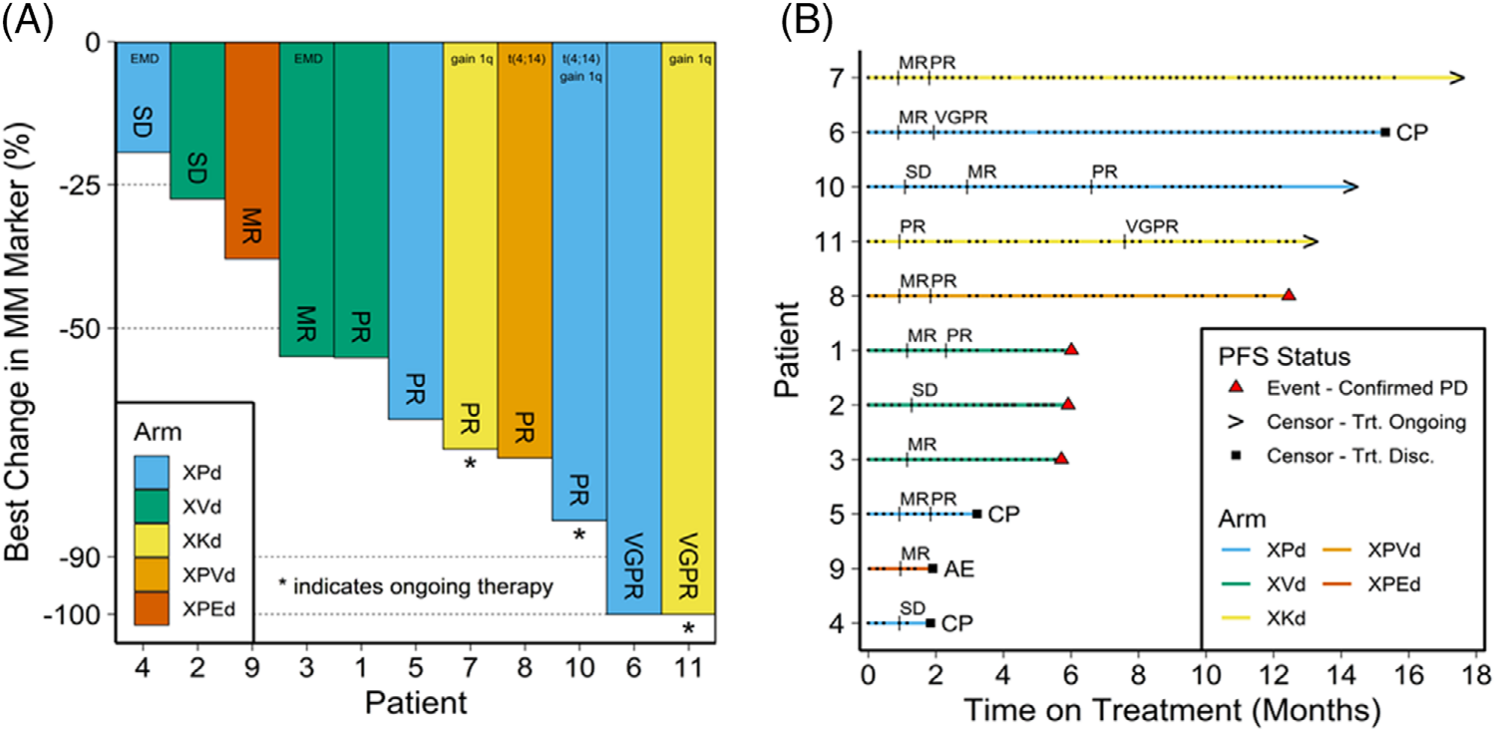
Treatment outcomes in response to X‐containing regimens. (A) Response of multiple myeloma (MM) markers to treatments. *Ongoing therapy at data cutoff (March 01, 2022). High‐risk cytogenetics defined as presence of del(17p), t(4;14), t(14;16), 1q+, or extramedullary myeloma at screening. Patient 3 with minor response (MR) also had an unconfirmed partial response (PR). (B) Swimmer plot of selinexor dosing and response assessments over time. Each point on each line of the swimmer plot represents dosing of selinexor. CP, clinical disease progression; d, dexamethasone; E, elotuzumab; EMD, extra medullary disease; K, carfilzomib; MR, minimal response; P, pomalidomide; PD, progressive disease; PR, partial response; SD, stable disease; V, bortezomib; VGPR, very good partial response; X, selinexor

Grade 3/4 AEs in ≥2 patients included thrombocytopenia (63.6%) without concurrent bleeding, neutropenia (45.5%), anemia (27.3%, all Grade 3), and lymphopenia (18.2%, all Grade 3). One patient treated with selinexor, pomalidomide, elotuzumab, and dexamethasone died of pulmonary nocardiosis considered related to all four study drugs. No new safety signals due to selinexor were reported. In prior studies of selinexor doublet or triplet combination regimens, infection‐related deaths attributed to the therapy were reported in 2 of 128 (1.5%) enrolled STORM patients treated with selinexor and dexamethasone (one with pneumonia and one with sepsis) [6], 6 of 195 (3.0%) BOSTON patients assigned to selinexor, bortezomib, and dexamethasone (three each with PNA and sepsis) [5], and none of the 32 patients treated with selinexor, carfilzomib, and dexamethasone triplet regime in STOMP [14] had any infection‐related deaths reported.

High rates of anti‐MM activity and tolerability of the XVd, X‐carfilzomib and dexamethasone, and X‐pomalidomide and dexamethasone triplets are described in heavily pretreated MM [5, 14, 15, 16]. The ORR, CBR, and PFS rates reported here with X‐containing regimens are numerically higher compared to those recently reported after non‐X‐containing therapy in the similar BCMA‐refractory space (albeit small comparative numbers) [12]. Nonetheless, this is promising for patients with anti‐BCMA refractory MM, is consistent with earlier reports [11], and with no evidence of cross‐resistance between X‐based and other MM regimens. This further reaffirms robust activity of X‐containing regimens in not only CAR‐T cell, but also ADC anti‐BCMA pretreated MM.

Taken together, among heavily previously treated patients, the majority with MM refractory to ADC (versus CAR‐T) anti‐BCMA therapy, X‐containing regimens had impressive potency with durable responses and ≥6 month tolerability. Notably, X‐containing regimens had an ORR and CBR higher than those with their prior anti‐BCMA therapies, despite their use of at least one treatment line later. Considering the emerging efficacy of X‐containing regimens in relapsed/refractory MM, including heavily pretreated anti‐BCMA subset, X‐containing combinations with novel IMiDs, PIs, or mAbs warrant investigation in earlier lines of therapy, including first relapse [11, 12, 14].

## AUTHOR CONTRIBUTIONS

MB conceived the work, analyzed the data, and wrote the manuscript. CG, GJS, SAT, NSC, SL, JM, RK, NJB, DW, CIC, HJS, DW, SM, RL, MS, CPV, WIB, NB, and BL collected the data. AD, DRVD, CZ, JJS, SS, MGK, and OSB contributed to study design and analyzed the data. All authors contributed to interpretation of the data. All authors edited, reviewed manuscript drafts, and approved the final version.

## CONFLICT OF INTEREST

Muhamed Baljevic ‐ Consultancy: BMS/Celgene, Cardinal Health; Sanofi‐Genzyme. Advisory Boards: Oncopeptides, Janssen Research, Karyopharm, BMS/Celgene, Sanofi‐Genzyme. Speaker: NCCN, CurioScience, AJH. Cristina Gasparetto ‐ Leadership: Celgene. Consulting or Advisory Role: Abbvie/Genentech; Celgene; GlaxoSmithKline; Janssen; Karyopharm Therapeutics; Sanofi. Speaker: GlaxoSmithKline; Karyopharm Therapeutics; Sanofi. Travel, Accommodations, Expenses: Celgene; Karyopharm Therapeutics. Gary J. Schiller ‐ clinical research support from KaryopharmSascha A. Tuchman ‐ Consulting: Caelum, Sanofi, Shattuck Labs, Janssen; Research support: Karyopharm, Sanofi, Caelum, Janssen. Natalie S. Callander ‐ Research Funding: Cellectar. Suzanne Lentzsch ‐ Leadership: Caelum Biosciences. Stock and Other Ownership Interests: Caelum Biosciences. Consulting or Advisory Role: Abbvie/Genentech; Amgen; Caelum Biosciences; Celularity; GlaxoSmithKline; Janssen; Sanofi; Sorrento Therapeutics; Takeda. Speaker: Clinical Care Options/NCCN; PeerView. Research Funding: Karyopharm Therapeutics; Sanofi. Patents, Royalties, Other Intellectual Property: Patent 11‐1F4 mAb for use in AL Amyloidosis. Travel, Accommodations, Expenses: Janssen. Jorge Monge ‐ Consultancy for BMS, Research funding from Karyopharm Therapeutics. Rami Kotb ‐ Research funding: Merck, Sanofi. Ownership/Share holder: Karyopharm. Honoraria: Celgene/BMS, Janssen, Takeda, Amgen, Sanofi, Merck, Pfizer. Nizar J. Bahlis ‐ Consultancy and advisory board: BMS/Celgene, Janssen, Pfizer, Amgen, Genentech, Sanofi, Karyopharm. Research funding: PfizerDarrell White‐ Amgen, Antengene, Celgene/BMS, Forus, GSK, Janssen, Karyopharm, Sanofi, Takeda: honoraria, consultancyChristine I. Chen ‐ Consulting or Advisory Role ‐ Abbvie; AstraZeneca; Bristol‐Myers Squibb; Gilead Sciences; Janssen; Novartis; Research Funding ‐ Gilead Sciences. Heather J. Sutherland ‐ Honoraria ‐ Amgen; Bristol‐Myers Squibb; Celgene; Genzyme; Janssen; Takeda. Consulting or advisory role ‐ Amgen; Bristol‐Myers Squibb; Celgene; Janssen; Sanofi; Takeda. Sumit Madan ‐ Speaker bureau: Janssen, BMS Ad hoc advisory board/consultancy: Janssen, Takeda, Oncopeptide, Pfizer. Richard LeBlanc‐ Consultancy/advisory board: BMS Canada; Janssen Inc.; Amgen Canada; Takeda Canada; Sanofi Canada. Michael Sebag ‐ Honoraria: Amgen; Bristol‐Myers Squibb; Celgene; Janssen‐Ortho; Karyopharm Therapeutics; Novartis; Takeda. Research funding: Janssen. Patents, Royalties, Other. Intellectual Property: Patent but with no associated royalties or profit. Christopher P. Venner ‐ Honoraria: Amgen; Bristol‐Myers Squibb; GlaxoSmithKline; Janssen; Sanofi; Takeda. William I. Bensinger ‐ Speakers bureau: Janssen, BMS, Amgen, Takeda, SanofiDane R. Van Domelen, Ohad S. Bentur, and Chris Zhang are employees of Karyopharm Therapeutics. Andrew DeCastro, Jatin J. Shah, and Michael G. Kauffman are former employees of Karyopharm Therapeutics. Sharon Shacham is a former employee of Karyopharm Therapeutics and holds patents (8999996, 9079865, 9714226, PCT/US12/048319, and I574957) on hydrazide‐containing nuclear transport modulators and uses and holding pending patents (PCT/US12/048319, 499/2012, PI20102724, and 2012000928) on hydrazide‐containing nuclear transport modulators and uses. Brea Lipe ‐ Consultant for BMS, Janssen, GSK.

## ETHICS STATEMENT

The study was approved and performed in accordance with the International Conference on Harmonization, the Guidelines for Good Clinical Practice, appropriate regulatory requirements, and with approval of institutional review boards at individual enrolling institutions. All patients provided written informed consent before study start.

## Data Availability

Karyopharm Therapeutics agrees to share individual participant data that underlie the results reported in this article (after deidentification), including the study protocol and statistical analysis plan. Data availability will begin 9 months after publication and will be available 36 months after publication. To gain access, data requestors should submit a proposal to medicalinformation@karyopharm.com. Proposals will be reviewed by an independent review committee identified for this purpose.
